# Association between diabetes and tuberculosis: case-control study

**DOI:** 10.1590/S1518-8787.2016050006374

**Published:** 2016-11-24

**Authors:** Susan Martins Pereira, Gleide Santos de Araújo, Carlos Antônio de Souza Teles Santos, Maeli Gomes de Oliveira, Maurício Lima Barreto

**Affiliations:** I Departamento de Saúde Coletiva. Instituto de Saúde Coletiva. Universidade Federal da Bahia. Salvador, BA, Brasil; II Programa de Pós-Graduação em Saúde Coletiva. Instituto de Saúde Coletiva. Universidade Federal da Bahia. Salvador, BA, Brasil; III Centro de Pesquisa Gonçalo Moniz. Fundação Osvaldo Cruz. Salvador, BA, Brasil; IV Departamento de Saúde. Universidade Estadual de Feira de Santana. Feira de Santana, BA, Brasil

**Keywords:** Diabetes Mellitus, Tuberculosis, Epidemiology, Comorbidity, Case-Control Studies

## Abstract

**OBJECTIVE:**

To test the association between diabetes and tuberculosis.

**METHODS:**

It is a case-control study, matched by age and sex. We included 323 new cases of tuberculosis with positive results for bacilloscopy. The controls were 323 respiratory symptomatic patients with negative bacilloscopy, from the same health services, such as: ambulatory cases from three referral hospitals and six basic health units responsible for the notifications of new cases of tuberculosis in Salvador, Bahia. Data collection occurred between 2008 and 2010. The instruments used were structured interview, including clinical data, capillary blood glucose (during fasting or postprandial), and the CAGE questionnaire for screening of abusive consumption of alcohol. Descriptive, exploratory, and multivariate analysis was performed using conditional logistic regression.

**RESULTS:**

The average age of the cases was 38.5 (SD = 14.2) years and of the controls, 38.5 (SD = 14.3) years. Among cases and controls, most subjects (61%) were male. In univariate analysis we found association between the occurrence of diabetes and tuberculosis (OR = 2.37; 95%CI 1.04–5.42), which remained statistically significant after adjustment for potential confounders (OR = 3.12; 95%CI 1.12–7.94).

**CONCLUSIONS:**

The association between diabetes and tuberculosis can hinder the control of tuberculosis, contributing to the maintainance of the disease burden. The situation demands increasing early detection of diabetes among people with tuberculosis, in an attempt to improve disease control strategies.

## RESUMO

**OBJETIVO:**

Testar a associação entre diabetes e tuberculose.

**MÉTODOS:**

Trata-se de estudo caso-controle, pareado por idade e sexo. Foram incluídos 323 casos novos de tuberculose com resultados positivos à baciloscopia. Os controles foram 323 sintomáticos respiratórios com baciloscopia negativa, oriundos dos mesmos serviços de saúde dos casos: ambulatórios de três hospitais de referência e seis unidades básicas de saúde responsáveis pelas notificações dos casos novos de tuberculose em Salvador, Bahia. A coleta de dados ocorreu entre 2008 e 2010. Os instrumentos utilizados foram entrevista estruturada, incluindo dados clínicos, glicemia capilar (em jejum ou pós-prandial) e o questionário CAGE para triagem de consumo abusivo de álcool. Foi realizada análise descritiva, exploratória e multivariada utilizando-se de regressão logística condicional.

**RESULTADOS:**

A média de idade dos casos foi 38,5 (DP = 14,2) anos e dos controles, 38,5 (DP = 14,3) anos. Tanto entre os casos quanto entre os controles, a maioria (61%) dos indivíduos era do sexo masculino. Na análise univariada, houve associação entre ocorrência de diabetes e de tuberculose (OR = 2,37; IC95% 1,04–5,42), que permaneceu estatisticamente significante após ajuste pelos potenciais confundidores (OR = 3,12; IC95% 1,12–7,94).

**CONCLUSÕES:**

A associação entre diabetes e tuberculose pode dificultar o controle da tuberculose, contribuindo para manutenção da elevada carga da doença. A situação demanda intensificação da detecção precoce de diabetes entre pessoas com tuberculose, na tentativa de maior efetividade das estratégias de controle da doença.

## INTRODUCTION

Tuberculosis (TB) is a neglected disease of great magnitude and transcendence and it has a close relationship with poor living conditions and social inequalities^20^ ^,^ ^27^. The risk is greater in vulnerable socioeconomic groups and people with other diseases, such as HIV, alcoholism, chronic lung diseases, cancer, malnutrition, and diabetes^14^ ^,^ ^19^ ^,^ ^22^.

The possibility of association between diabetes mellitus (DM) and TB represents an important and growing challenge to the global control of TB^7^ ^,^ ^27^. Patients with these two conditions may present high rates of treatment failure of TB and increased risk of death^8^ ^,^ ^19^. Cases of TB with DM have higher probability of treatment failure of TB and may develop resistance to the drugs used in the treatment. On the other hand, TB can induce glucose intolerance and hinder the glycemic control in individuals with DM^8^ ^,^ ^12^.

The global prevalence of DM was estimated at 8.3% (382 million of adults) in 2013, with projection of an increase to 8.8% (592 million of adults) in 2035^9^. It is estimated, in 2015, about 5 million deaths in the world due to this disease that affects all age groups, with a predominance of the group between 45 and 64 years^9^ ^,^ ^a^. Despite being one of the most common chronic diseases in almost all countries, only 50.0% of individuals affected are aware of the clinical diagnosis of DM, which compromises its control^a^ ^,^ ^b^.

In Brazil, a population-based multicenter study, conducted in nine capitals estimated the prevalence of DM in adults at 7.6% in 1998. In 2015, the estimative was 10.2% (14.3 million of adults between 20-79 years), with perspective for an increase in the epidemy^13^ ^,^ ^a^. Study on the predictors of treatment outcome identified that the tuberculosis mortality rate was 6.1%; for that group, the diabetics showed increased risk of mortality (RR = 3.94)^19^.

This picture adds to the high TB endemicity in Brazil, which occupies the 16^th^ place in the ranking of the 22 highest burden of disease in the world^26^. In 2014, 67,966 new cases of TB were diagnosed, with a coefficient of incidence of 33.5/100 thousand inhabitants. The distribution is heterogeneous among the Brazilian states and associated with social inequalities. Bahia is considered, by the National Strategic Plan for Tuberculosis Control, one of the priority states to implement public policies for the control of the disease. In 2014, the incidence of TB in Bahia was 30.7/100 thousand inhabitants. Its capital, Salvador, showed incidence of 62.7/100 thousand inhabitants, higher than the double of the number verified in the state that year^17^.

The growth of the obesity epidemic, the industrialization, urbanization, and the changes in lifestyle increase the prevalence of the DM^8^ ^,^ ^a^, with effect in TB, being considered the existence of confluent epidemics of DM and TB in developing countries^7^ ^,^ ^b^. The susceptibility to infections in DM occurs due to the decrease of cellular and humoral immunity and can lead to a resurgence of TB in endemic regions, especially in urban areas, which would entail a potential risk of expansion with serious implications for the control^5^ ^,^ ^8^. Recently, the subject led to research in several countries, with results that suggest DM as a risk factor for TB^6^ ^,^ ^16^. In Brazil, research on the subject is scarce and studies on the proportion of incident TB cases that can be attributed to the DM have not been identified. Thus, this research on the incidence of DM in TB is very important.

In Brazil, the morbimortality profile also changed. Infectious diseases are no longer the main causes of mortality; however, some of these diseases persist, such as TB, that, even showing improvements in some indicators of control, remains as a public health problem. Currently, in addition to the classic socioeconomic and demographic risk factors for TB, the convergence of non-infectious chronic health problems deserves attention^7^ ^,^ ^14^ ^,^ ^15^. This research aimed to test the association between DM and TB.

## METHODS

This case-control study includes incident cases of pulmonary TB, matched by age (with a variation of ± 5 years) and sex in the proportion 1:1. It was held in the city of Salvador (Bahia state, Northeastern Brazil), whose population is estimated at 2.94 million inhabitants in 2016 and territory of 692,819 km; it has a medium human development index (HDI), and the life expectancy at birth in the city increased from 69.6 years in 2000 to 75.1 years in 2010^c^ ^,^ ^d^. We included the main care centers of TB cases of the Brazilian Unified Health System (SUS): ambulatories from three referral hospitals and six basic health units. In addition to the TB being a notifiable disease in Brazil, its diagnosis and treatment are available for free on the public services network.

The sample size was calculated in 187 cases and 187 controls, considering 5% significance level, test power of 95%, odds ratio of 3.0 and proportion of DM exposure equal to 6.4%, equivalent to the one estimated for Brazil in 2010^e^.

New cases with suggestive clinical symptoms of pulmonary TB, which presented positive bacilloscopy and culture, over 15 years of age, participated in this study. The cases included resided in the city of Salvador and had no history of previous treatment for pulmonary TB. The controls were symptomatic respiratory patients who presented negative bacilloscopy and sputum culture, belonging to the same age group of the cases, residents in Salvador, without previous history of active pulmonary TB and from the same health units of the cases.

Data were collected by a team of trained nursing technicians under the supervision of nurses between August 2008 and April 2010. Cases and controls were interviewed when the patients were accessing the health service. After the exams’ results based on medical diagnosis, those who were diagnosed with a new case of TB were classified as cases. Those whose TB diagnosis had been excluded by the doctor were classified as controls. New cases of TB with previous diagnosis of the disease were excluded. The situation of the participant was monitored since the interview until the confirmation or exclusion of the diagnosis of pulmonary TB by the doctor.

For exams of bascilloscopy and culture, the sputum of cases and controls were obtained by expectoration, collected in sterile container, packed in coolers, and forwarded to the Central Public Health Laboratory. For capillary blood glucose tests, fingerstick was performed and the Breeze II^TM^ 1451 glucometer was used. It was alcohol. The outcome analyzed was TB, classified as yes or no, according to the positive or negative result of the laboratory examinations. The main independent variable, DM, was categorized as yes and no, using the result of capillary blood glucose during fasting or postprandial. The cut point of blood glucose values for DM diagnosis was based on the guidelines of the American Diabetes Association (fasting blood glucose ≥ 126 mg/dl or postprandial ≥ 200 mg/dl)^1^.

The sociodemographic covariates were monthly family income (≤ 1 minimum wage; > 1 minimum wage), marital status (married; stable; other) type of residence (purchased; rented; other) history of contact with someone with TB (yes; no), years of study (0-4 years, ≥ 5 years), skin color (white; mixed-race; black; other) agglomeration (> 2 people/room; 0-2 people/room). For the socioeconomic variable, we used a score, from the number of goods and household items (radio; television; fridge; DVD; microwave; computer; washing machine; cell phone; telephone; stove; video camera recorder; car), two categories were considered: low material comfort (0-7 items) and great material comfort (8-12 items). Sociodemographic variables and life habits were considered confounding variables, because they associate both with the exposure (DM) and with the outcome (TB). Alcoholism was defined by two positive responses (out of four) to the CAGE (positive; negative). Other variables related to life habits were drug use (yes; no) and smoking (yes; no).

Exploratory analysis was used to evaluate the distribution of the variables, the presence of lost data, and inconsistencies. Bivariate analysis was conducted using the Chi-square test, considering the significance level of 5%. The association was measured by odds ratio (OR) and their respective 95% confidence intervals. In multivariate analysis, progressive conditional logistic regression was used using the backward method to eliminate the variables of the complete model. The variables that showed association with the outcome (p ≤ 0.25) in uni and multivariate analysis were included in the model; epidemiological criteria were also considered and that is why the variable years of study remained in the final model, associated with TB and DM in other publications on the subject. The population attributable fraction (PAF) was estimated considering the studies directed by Levin et al., which uses the proportion of the exposed among the cases for PAF calculation^4^. All analyses were carried out using the version 12 of the Stata program.

This study was approved by the Research Ethics Committee of the Instituto de Saude Coletiva of the Universidade Federal da Bahia (Register 012-07/CEP-ISC). All participants signed the informed consent form in accordance with the guidelines stipulated in the Resolutions 196/96 and 304/2000.

## RESULTS

A total of 323 cases of TB and 323 controls were included. No differences were found between cases and controls regarding the matching variables. The average age was 38.5 years for cases and controls. Comparing the groups, males predominated (61%) (Table 1).

**Table 1:** Distribution of the matching variables between TB cases and controls. Salvador, BA, Northeastern Brazil, 2008-2010.

Variable	Cases (n = 323)			Controls (n = 323)			P
	
	n	%	Mean (SD)	n	%	Mean (SD)	
Sex							
Male	197	61.0		196	60.7		
Female	126	39.0		127	39.3		0.94
Age group							
15-29	93	28.8		96	29.7		
30-39	81	25.1		79	24.5		
40-49	75	23.2		69	21.3		
≥ 50 years	74	22.9		79	24.5		0.92
Age, in years			38.5 (14.16)			38.5 (14.34)	

The frequency of DM was higher among TB cases than among controls. Positive association was observed between having DM and presenting TB (crude OR = 2.86; adjusted OR = 2.86). The proportion of TB cases attributed to the DM was estimated at 8.5% (Table 2).

**Table 2:** Models of association between tuberculosis and diabetes mellitus adjusted for several covariates, for conditional logistic regression. Salvador, BA, Northeastern Brazil, 2008-2010.

Variables	Cases		Controls		OR_crude_	OR^1^_adjusted_	OR^2^_adjusted_	PAF (%)

	(n = 323)		(n = 323)					
Diabetes	n	%	n	%				
Yes	44	13.6	18	5.6	2.68 (1.55–5.25)	2.65 (1.47–4.80)	2.65 (1.47–4.76)	8.5
No	279	86.4	305	94.4				

PAF: population attributable fraction

OR^1^: Adjusted for all variables (household income, marital status, type of residence, history of contact with someone with TB, skin color, agglomeration, possession of goods and utensils, smoking, years of study, consumption of drugs, alcoholism); OR^2^: Adjusted for alcoholism, family income, years of study, agglomeration.

## DISCUSSION

This is the first study conducted in Brazil to investigate the association between DM and TB using primary data. The proportion of TB cases among the carriers of DM was elevated (13.6%), higher than that found in other underdeveloped or developing countries, such as Nigeria (5.7%)^18^, India (8%)^10^, Peru (11.1%)^12^, and China (12.0%)^16^. The magnitude of the association between diabetes and TB was compatible with the world literature, which shows variation between 2.44 and 8.33^7^ ^,^ ^16^. Recent meta-analyses have estimated that the presence of DM triples the risk of TB^11^ ^,^ ^b^.

Associations of similar magnitudes have also been reported in countries where TB is not considered to be endemic, such as the United States^5^ and Australia^6^. These findings may interfere in the control of tuberculosis, as evidences^6^ ^,^ ^7^ ^,^ ^12^ ^,^ ^16^ show that DM, besides being a risk factor for the occurrence of TB, raises the possibility to reactivate latent cases. In addition, patients with DM and TB may delay the negativity of bacilloscopy after treatment initiation. Thus, it is necessary to consider the higher possibility of complications and increased risk of relapse of TB in regions with high prevalence of diabetes^8^ ^,^ ^12^.

The estimative is that it should occupy the fifth position in the world in relation to the number of diabetic adults in 2035, which raises the relative contribution of DM for the TB burden^21^. The joint occurrence of DM and TB hinders the control of both diseases^23^ and may represent an important financial burden to public health.

Nevertheless, the impact of DM on the incidence of TB in this study was lower than that observed in other investigations. In Asia, the proportion of incident TB cases that can be attributed to the DM (evaluated by PAF) was elevated both in men (19.6%; 95%CI 10.9–33.1) and women (14.2%; 95%CI 7.1–26.5)^25^. In India, this impact on the total of TB cases was 15.8%^23^. Systematic review of the literature estimated that, in 2030, in the 10 countries with the highest burden of TB, 12.6% (95%CI 9.2–17.3) of the new cases of TB should be attributed to DM^21^.

Unlike the expected in the process of epidemiological transition, TB and other infectious diseases maintained the high prevalence in many of the developing countries, enabling the coexistence of infectious diseases with chronic-degenerative health problems, which increased in the last decades^14^ ^,^ ^23^. DM and TB affect vulnerable groups, such as older adults and people with other morbidities (hypertension, respiratory diseases, mental disorders, cancer)^3^ ^,^ ^14^ ^,^ ^23^. Diabetes contributes to the occurrence of unfavorable outcomes in the treatment of TB^7^ ^,^ ^16^, showing the need for special attention to the health of these groups.

In this study, the possibility of memory bias is minimized, because the individuals were interviewed when seeking initial care at health services due to respiratory symptoms, characteristic of TB, regardless of the diagnostic confirmation. As for the selection bias, the cases and controls were classified according to medical diagnosis; in addition, the auxiliary examinations of bacilloscopy and culture reduced the possibility of false positives and false negatives^2^. To identify individuals with DM, capillary blood glucose was used due to its convenience, fast and safe results, although this test is not the gold standard for diagnosis of DM. This method is suitable for use in screening of glucose changes, possessing high performance (precision and accuracy) in comparison with glucose^24^.

The findings of this study point to another challenge for the control of TB. It is possible that, despite their importance, the traditional existing measures that do not consider the convergence between DM and TB are not sufficient. In this context, the planning of integrated actions must be considered to confront the disease dual burden, as well as its consequences for individuals and health services.

## Footnotes

a International Diabetes Federation. [homepage na internet]. Atlas country summary table: estimates for 2015 Seventh Edition. [cited 2016 Oct 25]. Available from: http://www.diabetesatlas.org/

b World Health Organization. Collaborative framework for care and control of tuberculosis and diabetes. Geneva: World Health Organization; 2011 [cited 2015 Jul 25]. Available from: http://apps.who.int/iris/ bitstream/10665/44698/1/ 9789241502252_eng.pdf

c Instituto Brasileiro de Geografia e Estatística. Estimativas de população para 1º de julho de 2015: Cidades: Bahia. Rio de Janeiro: IBGE; 2015 [cited 2016 Oct 28]. Available from: http://www.ibge.gov.br/home/estatistica/populacao/estimativa2015/estimativa_tcu.shtm

d Salvador. City Hall Municipal Secretariat of Health Municipal Health Plan 2014-2017. Salvador: City Hall; 2014 [cited 2015 July 25]. Available from: http://www.saude.salvador.ba.gov.br/arquivos/astec/pms_2014_2017_versao_preliminar.pdf

e International Diabetes Federation. Prevalence estimates of diabetes mellitus (DM), 2010 – SACA. Brussels: International Diabetes Federation; 2010 [cited 2015 Jan 15]. Available from: http://www.idf.org/content/saca
